# Supplementary material for: Integrated population clustering and genomic epidemiology with PopPIPE
Source: Microb Genom. 2025 Apr 28;11(4):001404. doi: 10.1099/mgen.0.001404 (PMC12038005; doi:10.1099/mgen.0.001404)
Supplement: Uncited Supplementary Material 1. [file mgen-11-01404-s001.pdf]

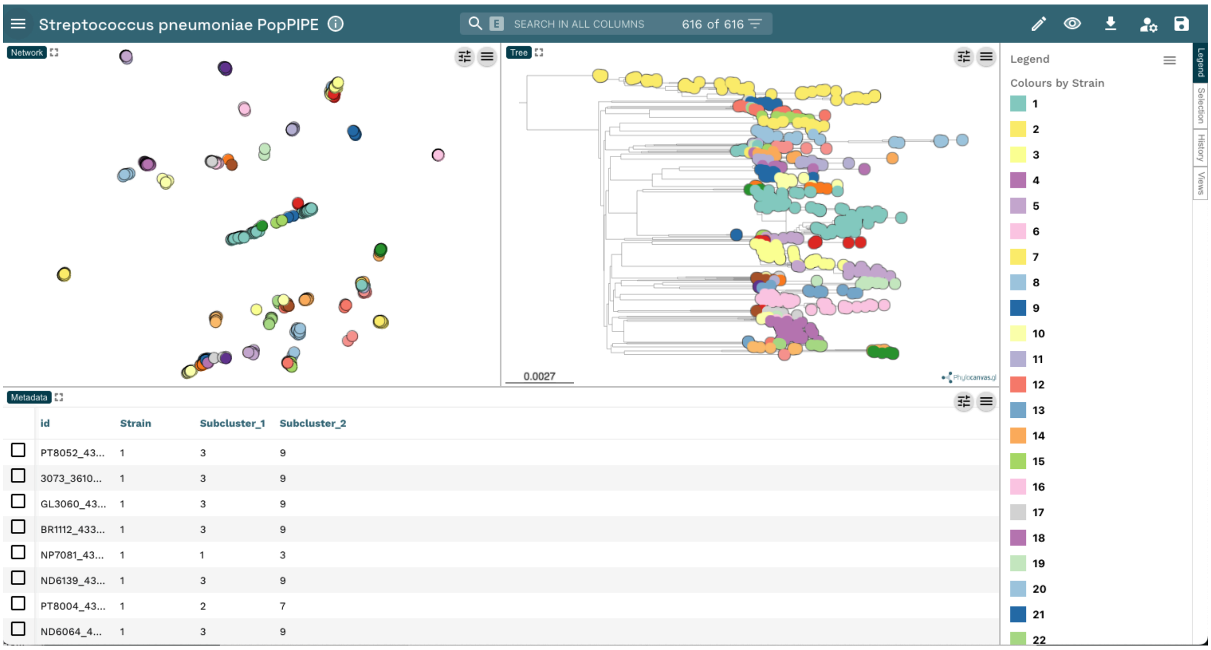

Supplementary figure 1: Full visualisation of the PopPIPE run from figure 5. Also available online: https://microreact.org/project/sparc-poppipe

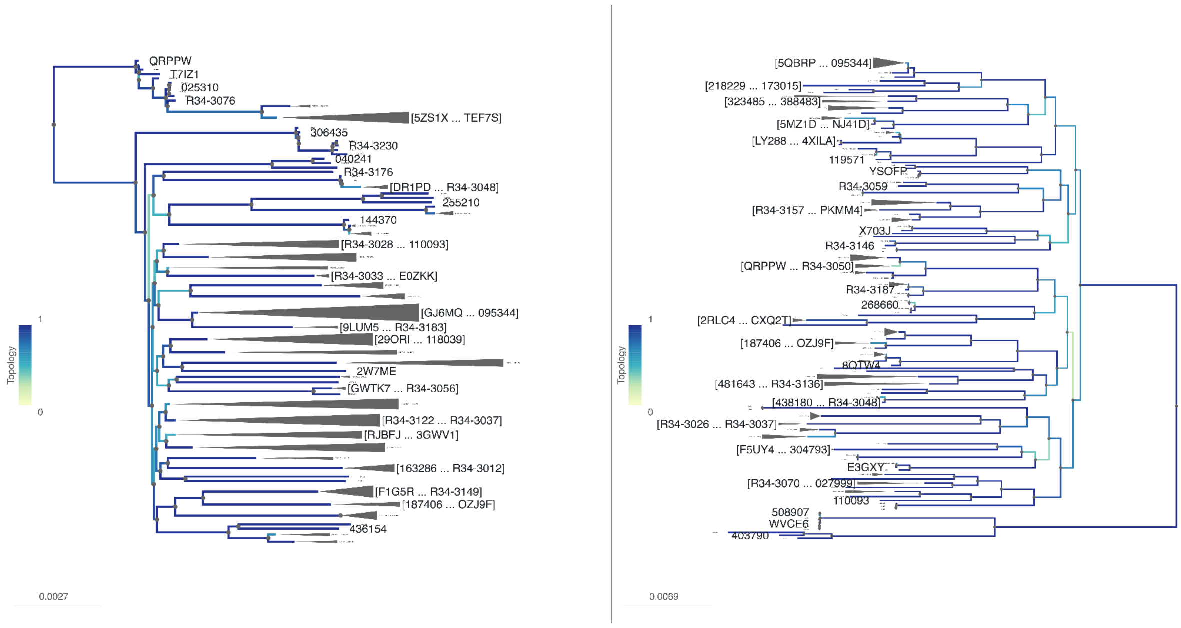

Supplementary figure 2: Comparison of original core-genome analysis and PopPIPE results in phylo.io. Files to recreate this view are available on figshare: 10.6084/m9.figshare.28429574.

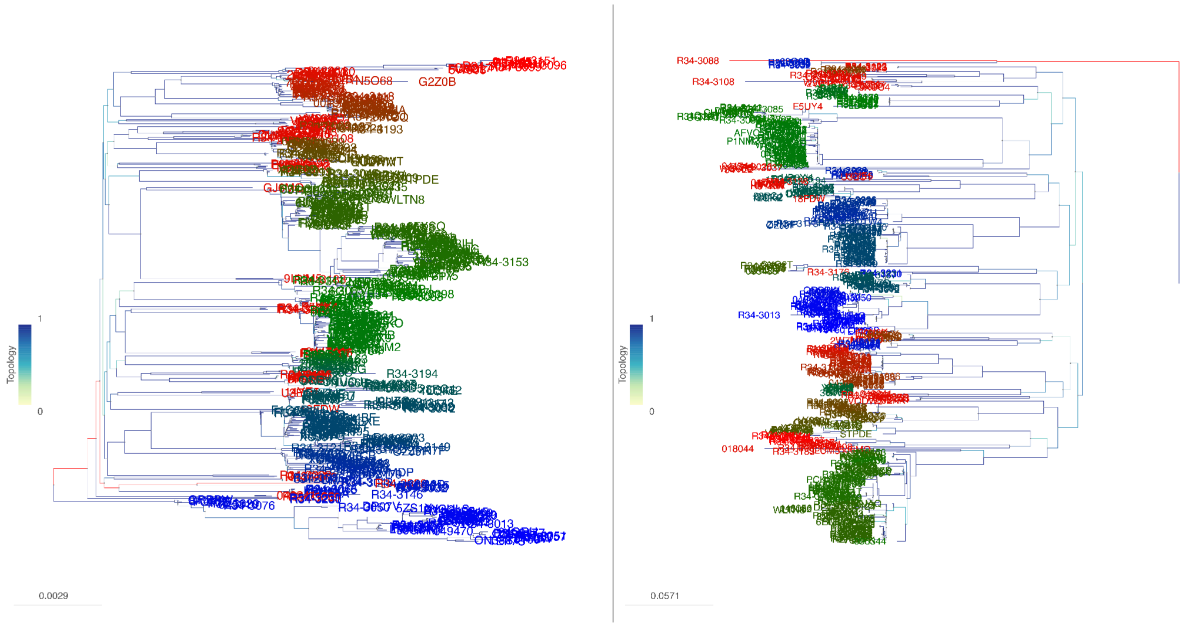

Supplementary figure 3: Comparison of original core-genome analysis and PopPIPE results in phylo.io including subcluster labels. Files to recreate this view are available on figshare: 10.6084/m9.figshare.28429574.

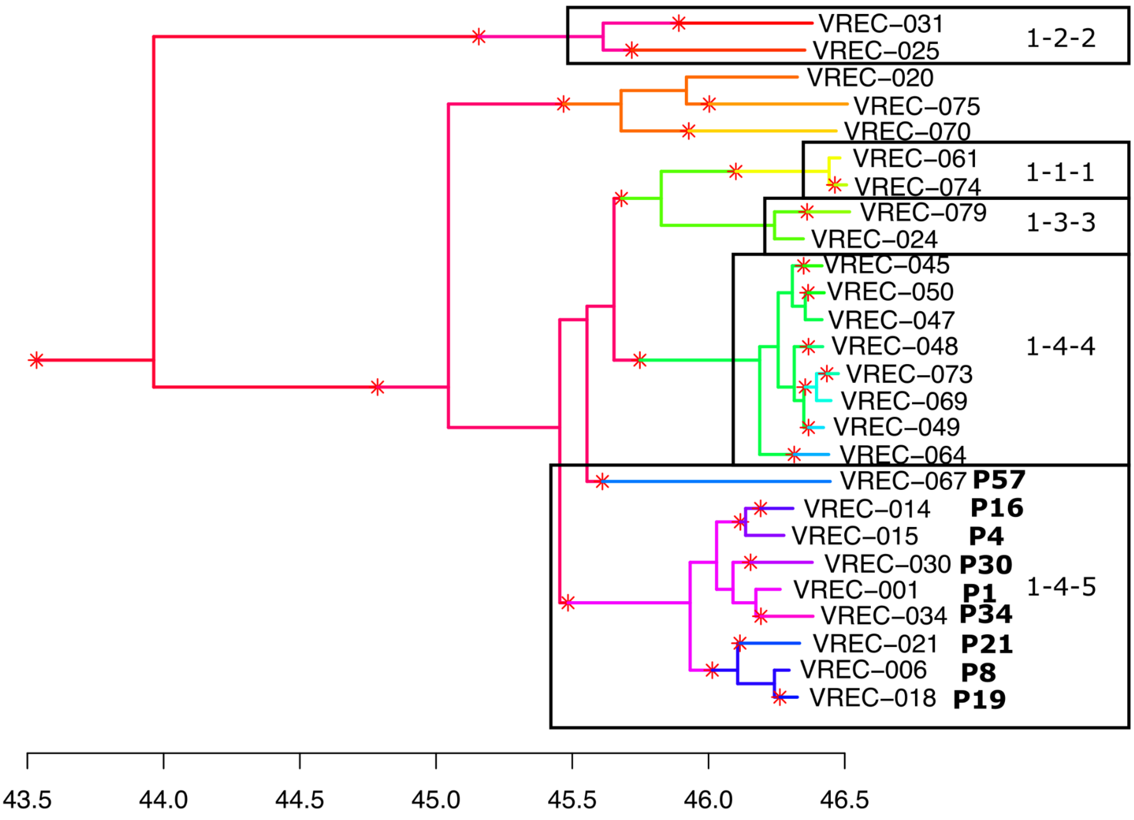

Supplementary Figure 4: TransPhylo transmission tree for PopPUNK cluster 1. PopPIPE clusters are indicated on the tree, and patient IDs have been added for cluster 1-4-5. Branches are coloured for each host, including inferred unsampled hosts, changes of colour on branches correspond to inferred transmission events from one host to another.
